# PCNA interacts with Prox1 and represses its transcriptional activity

**Published:** 2008-11-17

**Authors:** Xiaoren Chen, Tapan P. Patel, Vladimir I. Simirskii, Melinda K. Duncan

**Affiliations:** Department of Biological Sciences, University of Delaware, Newark, DE

## Abstract

**Purpose:**

Prox1 is a transcription factor which can function either as a transcriptional activator, transcriptional repressor or a transcriptional corepressor. This paper seeks to better understand the role of protein–protein interactions in this multitude of functions.

**Methods:**

We performed a yeast two-hybrid screen of an 11.5 day post coitum (dpc) mouse embryo cDNA library using the homeo-Prospero domain of Prox1 as bait. Computer modeling, cotransfection analysis and confocal immunolocalization were used to investigate the significance of one of the identified interactions.

**Results:**

Proliferating cell nuclear antigen (PCNA) was identified as a Prox1 interacting protein. Prox1 interactions with PCNA require the PCNA interacting protein motif (PIP box), located in the Prospero domain of Prox1. Computer modeling of this interaction identified the apparent geometry of this interface which maintains the accessibility of Prox1 to DNA. Prox1 activated the chicken βB1-crystallin promoter in cotransfection tests as previously reported, while PCNA squelched this transcriptional activation.

**Conclusions:**

Since PCNA is expressed in the lens epithelium where Prox1 levels are low, while chicken βB1-crystallin expression activates in lens fibers where Prox1 expression is high and PCNA levels are low, these data suggest that Prox1-PCNA interactions may in part prevent the activation of βB1-crystallin expression in the lens epithelium.

## Introduction

Prox1 is a transcription factor necessary for the development of diverse organs including the lens, retina, liver, pancreas, inner ear, and lymphatic endothelium [1-6]. Prox1 has been proposed to be a tumor suppressor in hepatocytes [7] although it induces proliferation of fetal hepatoblasts [8]. Upregulation of Prox1 expression induces the progression of colon cancer [9] and the invasiveness of Karposi’s sarcoma [10] while the overexpression of Prox1 in blood endothelial cells induced their conversion to a lymphatic endothelial phenotype associated with upregulation of cell proliferation markers including PCNA, cyclin E1 and E2 [11]. However in the lens, loss of Prox1 function led to down-regulation of the cell cycle inhibitors P27*^KIP1^* and P57*^KIP2^*and a loss of lens fiber cell differentiation [1]. Thus, it appears that Prox1 may function in both cell cycle arrest and cell cycle progression depending upon the cellular environment.

The carboxyl-terminal DNA binding domain of Prox1 is well conserved within the animal kingdom and consists of an atypical homeodomain linked to a stretch of evolutionarily conserved amino acids denoted the Prospero domain [12] although crystallographic studies of its *Drosophila* counterpart, Prospero, suggest that the homeo- and Prospero domains fold into a single structural unit [13]. The amino-terminus of Prox1 interacts with HDAC3 to mediate its function as a transcriptional repressor [14]. Prox1 also has three nuclear receptor boxes (NR box) which can participate in Prox1 interactions with nuclear hormone receptors [15]. The subcellular localization of Prox1 has been proposed to be controlled by competition between a nuclear localization signal (NLS) located at the beginning of the amino-terminus and a nuclear export signal (NES) located in front of the homeodomain [16].

Prox1 activates the transcription of the chicken βB1-crystallin [17] and FGF receptor 3 [18] promoter via direct promoter interactions, although interaction of Prox1 with two of the three known binding sites in the βB1-crystallin promoter leads to transcriptional repression in cotransfection studies [19]. Prox1 also activates the mouse γF crystallin [20] and mouse cyclin E promoters [11] although direct promoter interactions were not demonstrated. Prox1 serves as a transcriptional corepressor of the nuclear hormone receptors LRH-1 [14,15], HNF4α [21] and SF-1 [22] via interactions of these factors with the nuclear receptor boxes of Prox1. In addition, Prox1 can to function as a direct DNA binding transcriptional repressor [6,19]. These data demonstrate that Prox1 is a multifunctional transcription factor whose function is likely to be modulated by protein–protein interactions.

To identify additional Prox1 interacting proteins which affect Prox1 function, we created a yeast two-hybrid prey vector containing the evolutionarily conserved carboxyl-terminal homeo-Prospero domain of Prox1 and screened an 11.5 day post coitum (dpc) mouse embryo cDNA library. We identified 15 possible Prox1 interacting proteins including the cell cycle related protein, proliferating cell nuclear antigen (PCNA). PCNA is best known as a sliding platform that stabilizes the interaction of other proteins with DNA during DNA replication and DNA repair and the coordination of these processes with the cell cycle [23]. Most reported interactions with PCNA are mediated via a conserved PCNA interacting protein motif (PIP box) found in PCNA interacting proteins [23]. Notably, such a motif is present in the Prospero domain of vertebrate Prox1. We found that Prox1 interacted with both the carboxyl-terminal domain and the IDCL (interdomain connecting loop) of PCNA and that mutation of the PIP box found in Prox1 diminished the interaction. In cotransfection studies, PCNA repressed Prox1 mediated activation of the βB1-crystallin promoter in transfection assays, indicating that PCNA negatively regulates Prox1 function. This is consistent with other reports that PCNA interacts with transcription factors and represses their transcriptional activity [24-26]. Since PCNA is expressed in proliferating lens epithelial cells, while it is downregulated sharply early in lens fiber cell differentiation, it is possible that PCNA modulates Prox1 mediated fiber cell differentiation in lens epithelial cells.

## Methods

### Yeast two-hybrid analysis

A yeast two-hybrid screen bait plasmid was constructed by cloning a PCR generated cDNA fragment corresponding to the homeo (HD) and Prospero (PD) domain (amino acids 547–737), of human Prox1 into the EcoRI/BamHI site of pGBKT7 (Clonech, Palo Alto, CA) to create fusion proteins between these Prox1 fragments and the DNA binding domain of yeast Gal4. Although this vector caused some autoactivation of the nutritional selection markers when transformed into yeast strain AH109, these yeast did not survive under high stringency selection. Similarly, yeast created by mating this strain with strain Y187 yeast harboring a vector expressing a fusion between the Gal4 activation domain and SV40 T-antigen also did not survive the high stringency selection, indicating that the autoactivation by Prox1 would not interfere with our screen (data not shown).

An 11 dpc mouse embryo oligo (dT) primed cDNA library cloned into the GAL4 activation domain (AD) vector pACT2 and pretransformed into yeast strain Y187 (1X 10^6^ primary clones) was obtained from Clontech. Strain AH109 yeast containing the pGBKT7-Prox1 HDPD construct was mated with the library strain and the primary transformants transferred to screening plates (SD/-Leu/-His/-Ade/-Trp with added X-α-Gal) using the Matchmaker 3 high stringency screening protocol (Clontech). The phenotype of the positive colonies was confirmed by restreaking onto another high stringency plate and the activation domain plasmids from positive colonies were rescued and sequenced.

Prox1-Gal4 DNA binding domain fusion constructs consisting of either HD (amino acids 547–642) alone or the PD (amino acid 638–737) alone of Prox1 were generated by ligating PCR generated cDNA fragments into pGBKT7. Site directed mutants in the PIP box (amino acid 686 to 693) [23] of human Prox1 (amino acid 686 Q to E/A; amino acid 689 L to A; amino acid 692 F to A and amino acid 693 F to A) [27] were created with the Quick Change site directed mutagenesis kit (Stratagene, La Jolla, CA) using the homeo-Prospero domain/pGBKT7 construct as a template. All constructs were sequence confirmed then transformed into yeast strain AH109.

The rescued AD/library plasmid containing PCNA was transformed into yeast strain Y187 which was then mated to yeast strain AH109 containing bait plasmids harboring either Prox1 constructs consisting of the HD/PD, HD, PD or HD/PD with PIP box mutations and transferred onto SD/-Leu/-Trp/X-α-Gal plates. The presence of PCNA-Prox1 interactions were tested by streaking the resulting colonies on both high and low stringency media and the relative strength of the interactions tested by β-Galactosidase assays using ONPG as substrate as described in the yeast protocols handbook (Clontech).

Mouse PCNA full length cDNA was synthesized by RT–PCR using newborn mouse lens total RNA. All PCNA truncations were made by PCR using full length PCNA as a template, including the N+ (amino acids 1–135); C+ (amino acids 115–261) and C- (amino acid 135–261). The products were digested with EcoR I and BamHI, and then ligated in frame into the pGADT7 vector (Clontech). All constructs were confirmed by DNA sequencing. The plasmids were then transformed into yeast strain Y187 and mated with AH109 containing the bait plasmids.

### Constructs

The 3XPL2-βActin CAT and -432/+30 pBasicCAT [28] and CMV human Prox1 [20] expression vector were previously described. pCMVβGAL was purchased from Clontech. The pCAT-Basic plasmid was obtained from Promega (Madison, WI). Full length mouse PCNA cDNA was ligated into the EcoR I/BamH I sites of the pcDNA3.1 vector (Invitrogen, Carlsbad, CA).

### Computer modeling of PCNA-Prox1 interactions

The X-ray crystal structure of full length human PCNA was retrieved from the Protein Databank (PDB accession code 1vym). The crystal structures of both apo- and DNA bound homeo-Prospero domain of *Drosophila* Prospero (PDB accession codes 1mij and 1xpx) [13,29] were used as knowledge based homology modeling templates to predict the structure of amino acids 580–727 of chicken Prox1 (100% identical in this region to the human sequence) as previously described [19]. The predicted secondary-structure of the homeo-Prospero domain of Prox1 is very similar to that of *Drosophila* Prospero and the Ramachandran plot for the Prox1 homology model is very similar to the known Prospero structure in this region [19].

The protein docking algorithm, ZDOCK, which optimizes desolvation, grid-based shape complementarity and electrostatics using a Fast Fourier Transform algorithm to generate plausible protein-ligand poses [30], was used for the initial-stage docking of Prox1 and PCNA . In the filtering stage, ClusPro, which ranks the complexes based on their desolvation and electrostatic energy, was used for subsequent ranking and refinement. The pair wise binding site root mean squared deviation was used to cluster the structures and the top three complexes were considered the most likely conformations of the PCNA-Prox1 complex [31,32]. The interactions between the homeo-Prospero domain of Prox1 and the isolated carboxyl-terminus of PCNA lacking the interdomain connecting loop were also modeled since this reflects the initial interaction identified in the yeast two-hybrid screen. Both predicted models were subjected to a short energy minimization cycle (2000 steps) using AMBER [33]. The minimized models were then compared by their percent buried surface area.

### Cell culture, transfection and reporter gene analysis

Chinese hamster ovary (CHO) cells [34] were cultured in Dulbecco's Modified Eagle Media (DMEM) supplemented with 10% fetal bovine serum at 37 C and 5% CO_2_. Transfections using CAT reporter plasmids were performed by plating the CHO cells (3.6x10^5^ cells) on a 60 mm dish followed by transfection the following day using Lipofectamine Plus (Invitrogen) with 0.25 μg of pCMV/β-GAL, 3 μg of promoter/CAT plasmidm and 0.25–1 μg of CMV expression plasmids. Cells were harvested 48 h after transfection and cellular extracts were prepared by multiple cycles of freeze/thaw. The extracts were assayed for CAT and β-galactosidase activity as previously described [19]. For transfection assays using luciferase reporter plasmids, CHO cells (1.2x10^4^ cells) were plated on 24 well plates the day before transfection and transfected with Lipofectamine plus with 0.01 μg pRL-TK, 0.35 μg of promoter/luciferase plasmid, and 0.02 μg of the CMV expression plasmids. The cells were collected 48 h after transfection and assayed for luciferase activity using the Dual-Luciferase^®^ Reporter (DLR™) Assay System (Promega, Madison, WI). All co-transfection experiments were performed at least twice in triplicate and analyzed statistically by Student’s *t* test.

### Immunohistochemistry

All experiments using animals received University of Delaware IACUC approval and conform to the ARVO statement on the use of animals in vision research. C57Bl6/N mice were produced in house and used for all experiments. Noon on the day that the vaginal plug was seen was defined as 0.5 dpc. Tissue was excised from these animals, embedded fresh in Optimum Cutting Temperature media (OCT, Tissue Tek, Torrance, CA), and 16 μm thick sections were prepared on a cryostat and mounted on ColorFrost plus slides (Fisher Scientific, Hampton NH). Sections were immersion fixed in 4% paraformaldehyde (PFA) for 5 min and in 95% ethanol for 10 min, blocked in SuperBlock blocking buffer (Thermo Scientific, Rockford, Illinois), and incubated for one hour at room temperature with a mixture of polyclonal goat anti-Prox1 (AF2727; R&D Systems, Minneapolis, MN) at a concentration of 4 μg/ml and rabbit polyclonal anti-PCNA (ab15497, Abcam, Cambridge, MA) at a concentration of 2 μg/ml. Following two 10 min washes, unlabeled primary antibodies were detected with mixture of appropriate Alexafluor 568 and Alexafluor 488 labeled secondary antibodies (Molecular Probes, Eugene OR) diluted in blocking buffer containing a 1:3000 dilution of the nucleic acid stain Draq-5 (Biostatus Limited, Leicestershire, UK). Slides were visualized with a Zeiss LSM 510 Confocal Microscope configured with an Argon/Krypton laser (488 nm and 568 nm excitation lines) and Helium Neon laser (633 nm excitation line) (Carl Zeiss Inc., Göttingen, Germany). All comparisons of staining intensity between specimens were done on sections stained simultaneously and the imaging for each antibody was performed using identical laser power and software settings to ensure validity of intensity comparisons.

## Results

### PCNA was identified as a Prox1 binding protein in a yeast two-hybrid screen

Prox1 expression begins in the mouse embryo around 7.5 dpc in the node [35] and is detected in the precursors of several Prox1 sensitive tissues including the pancreas, liver [35,36], and eye [1,37] by 9.5 dpc. By 11.5 dpc, Prox1 is expressed abundantly in the lens, liver, pancreas, and lymphatics and the first phenotypes are detected in Prox1 null mice [3,5,37,38]. Thus, we performed a yeast two-hybrid screen of an 11 dpc mouse embryo cDNA library to identify the binding partners of Prox1 during morphogenesis. This screen yielded 15 potential Prox1 interacting proteins including proliferating cell nuclear antigen (PCNA) with the original clone obtained consisting of the carboxyl-terminus of PCNA (amino acids 167–261).

**Figure 1:**
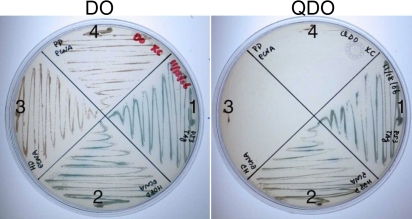
PCNA interacts with the integrated homeo-Prospero domain (HDPD) of Prox1 but not the homeodomain (HD) or Prospero domain (PD) alone. Yeast strain AH109 transformed with vectors expressing fusions of the DNA binding domain of GAL4 and the HDPD, HD, and PD, respectively, were mated with yeast strain Y187 transformed with a vector expressing the activation domain of GAL4 fused to the carboxyl-terminus of PCNA (original clone isolated from the library) and plated on double drop out (DO) media to select for the presence of both plasmids. Colonies from these plates were restreaked on either DO (to demonstrate presence of all vectors in the yeast) or quadruple drop out (QDO) plates to test for interaction between the protein products. (1) The known interaction between P53-SV40 T antigen was used as a positive control. (2) Yeast resulting from the mating between the HDPD bait plasmid and the PCNA prey plasmid grew on QDO plates and expressed the α-galactosidase reporter (blue colonies). (3) Yeast containing both the Prox1 HD and the C-terminus of PCNA did not grow under QDO selection. (4)  Yeast containing both the Prox1 PD and the C-terminus of PCNA did not grow under QDO selection.

Retransformation of the rescued PCNA clone into yeast containing the homeo-Prospero domain containing bait vector yielded yeast growth and blue colony formation on high stringency selection (quadruple drop out; QDO) plates demonstrating that this interaction occurs in yeast. In contrast, no growth was seen when the bait plasmid consists of the Gal4 DNA binding domain fused to the homeodomain or Prospero domain alone suggesting that it requires the integrated homeo/Prospero domain as defined by the Prospero crystal structure. (Figure 1).

**Figure 2:**
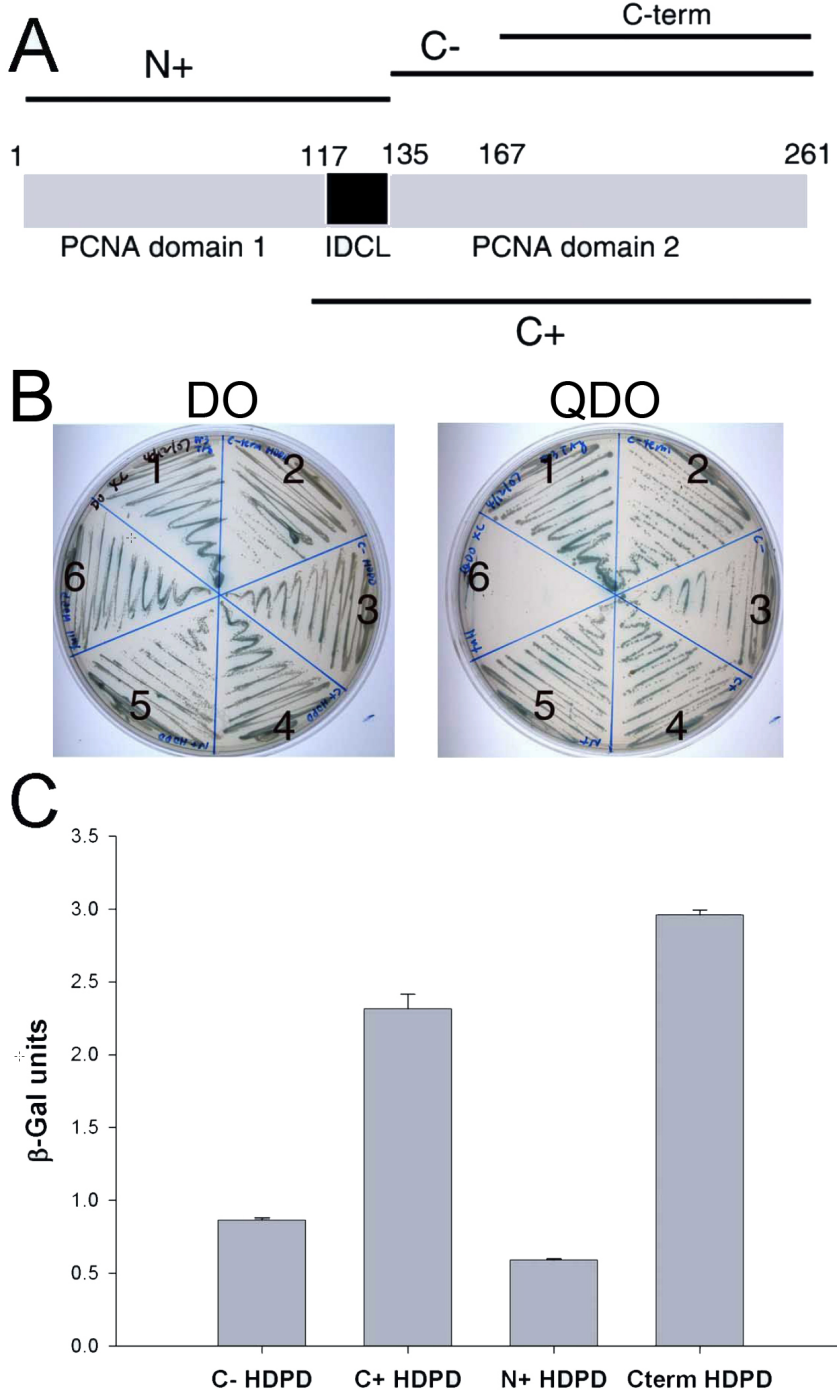
Prox1 interacted with the IDCL and the carboxyl-terminal domain of PCNA. **A**: Diagram of the structure of full length PCNA and the structure of the truncations used in this study. N+ (amino acids 1-135 of PCNA); C+ (amino acids 115-261 of PCNA); C- (amino acids 135-261 of PCNA); C-term, the original clone from the yeast two hybrid screen (amino acids 167-261 of PCNA). **B**: Yeast strain AH109 transformed with a vector expressing a fusion between the DNA binding domain of GAL4 and the HDPD of Prox1 was mated with yeast strain Y187 transformed with a vectors expressing either the activation domain of GAL4 fused to the C-term fragment of PCNA (2),  the C- fragment of PCNA lacking the IDCL (3), the C+ fragment of PCNA which includes the IDCL (4), the N+ fragment of PCNA (5) or full length PCNA (6). The interaction between P53 and SV40 large T-antigen was used as a positive control (1). Colonies from the original DO plates were re-streaked on either DO plates (to demonstrate presence of all vectors in the yeast) or quadruple drop out (QDO) plates to test for interaction between the protein products. **C**: QDO liquid cultures were grown of the yeast able to grow under QDO conditions and the relative amount of expression from the β-galactosidase reporter gene was determined by ONPG assays.

PCNA consists of three domains, the amino-terminal domain 1, the interdomain connecting loop (IDCL) and the carboxyl-terminal domain 2 (Figure 2A). We made a set of PCNA fusion constructs to map PCNA interactions with Prox1 and found that the homeo/Prospero domain was able to interact with PCNA fragments containing either amino acids 167–261 of the carboxyl-terminal domain two (the original clone), the entire carboxyl-terminal domain two, the carboxyl-terminal domain 2 plus the IDCL or the amino-terminal domain one plus the IDCL as assayed by the ability of yeast to grow on high stringency selection (Figure 2B). Quantitation of the relative strength of these interactions revealed that the original carboxyl-terminal PCNA fragment as well as the IDCL with the carboxyl-terminal domain 2 fragment interacted most efficiently with the homeo/Prospero domain of Prox1 (Figure 2C).

**Figure 3:**
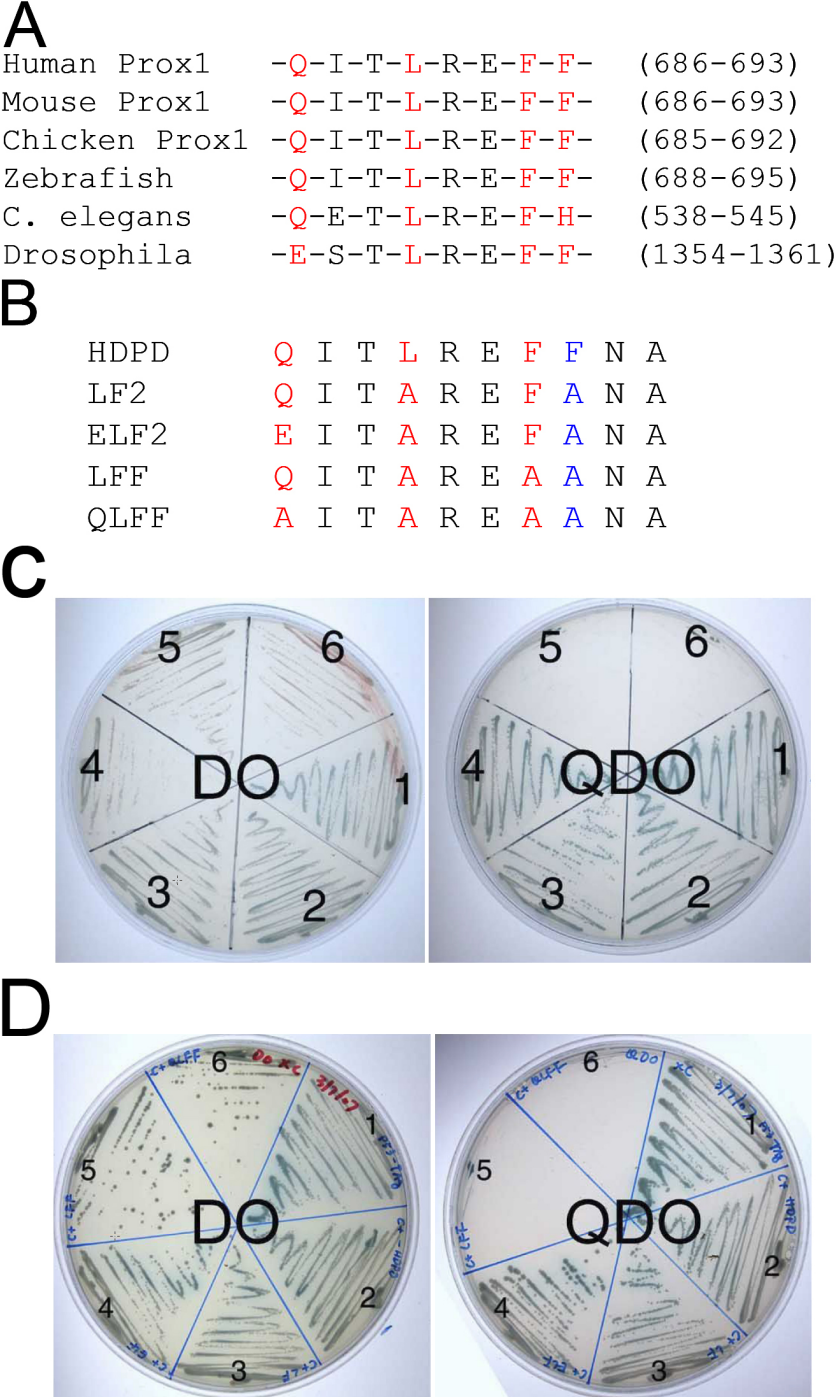
Prox1 interacted with PCNA through the PIP box. **A**: Sequence alignment of the PIP box-like sequence (red) in vertebrate Prox1 and its invertebrate homologs. **B**: The sequence of the PIP box found in Prox1 aligned with a series of mutants created in the HD/PD of Prox1 to test its role in PCNA-Prox1 interactions. **C**: The resulting PIP mutants were transformed into AH109 and mated with Y187 containing the original PCNA clone (amino acids 167-261). The work plates were made by restreaking the colonies obtained on the original DO plates on both DO (testing for the presence of the plasmids) and QDO plates (to test for protein-protein interactions). The wildtype HDPD interacted with PCNA (2) and the mutants LF (3) and ELF (4) still had the ability to bind. In contrast, mutants LFF (5) and QLFF (6) could not survive on QDO plates suggesting that they did not interact with the carboxyl-terminus of PCNA. The P53- SV40-T antigen interaction (1) was used as a positive control. **D**: The PIP mutants were transformed into AH109 and mated with Y187 containing the C+  PCNA clone which includes the IDCL (amino acids 115-261). The work plates were made by restreaking the colonies obtained on the original DO plates on both DO (to test for the presence of the plasmids) and QDO plates (to test for protein-protein interactions). The wildtype HDPD interacted with PCNA (2) and the mutants LF (3) and ELF (4) still had the ability to bind. In contrast, mutants LFF (5) and QLFF (6) could not survive on QDO plates suggesting that they did not interact with the carboxyl-terminus plus the IDCL of PCNA. The P53- SV40-T antigen interaction (1) was used as a positive control.

The IDCL of PCNA is known to interact with a wide variety of proteins which contain the consensus sequence Q-X-X-(I/L/M)-X-X-(F/Y)-(F/Y) denoted the PIP box [23]. This consensus was detected in the Prospero domain of vertebrate Prox1 while it is more divergent in worms and flies (Figure 3A). The importance of the PIP box of Prox1 for interactions with PCNA was tested by creating a series of Prox1 PIP box mutants by site-directed mutagenesis (Figure 3B). The mutants were transformed into yeast strain AH109 which was mated with yeast strain Y187 containing either the original PCNA clone consisting of the C-terminus of PCNA (Figure 3C) or the carboxyl-terminal domain two plus the IDCL (Figure 3D). All mating mixtures grew on low stringency medium (DO), indicating that the mating was successful. However, when these colonies were restreaked onto high stringency (QDO) plates, no growth was seen for mutants LFF or QLFF showing that the interaction between PCNA and the homeo-Prospero domain of Prox1 was abrogated (Figure 3C,D). This suggested that Prox1 interactions with PCNA require an intact PIP box in the Prospero domain.

### Modeling of Prox1-PCNA interactions

**Figure 4:**
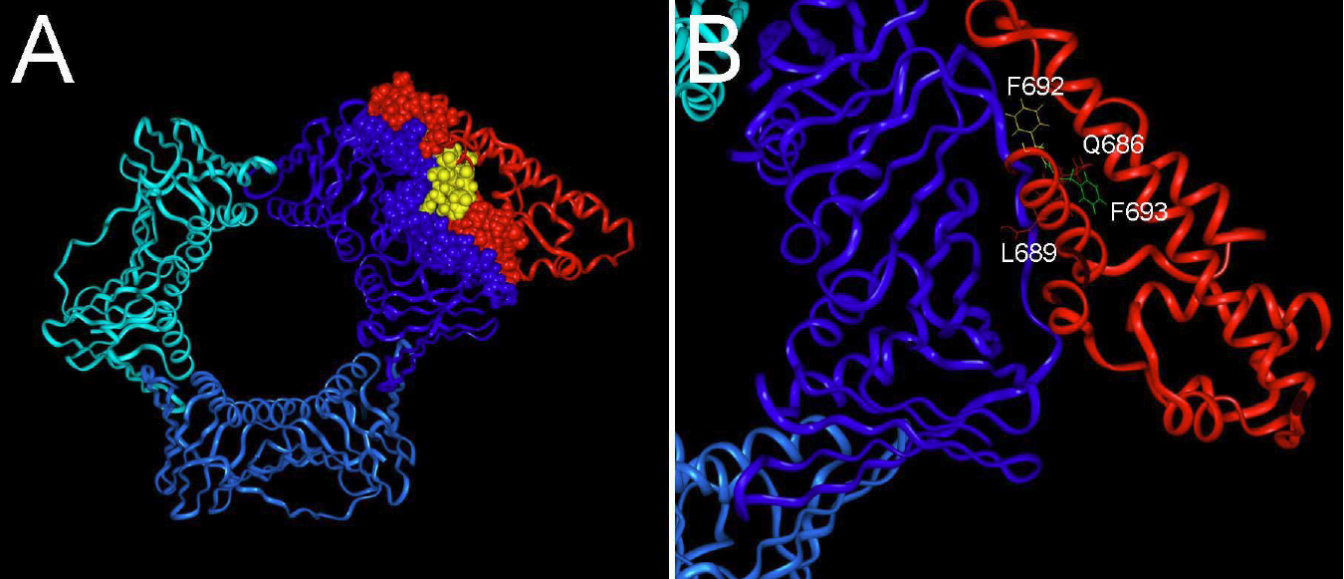
Docking models of Prox1-PCNA interaction. **A**: Ribbon diagram of the complete trimeric PCNA structure (blue) docked to the homeo-Prospero domain of Prox1 (red). Interface residues are depicted as van der Walls spheres and the PIP box of Prox1 is shown in yellow. **B**: Ribbon diagram of the interface between the IDCL of PCNA (blue) and Prox1 (red). Residue F693 (green) faces away from the surface and is inaccessible for interaction with PCNA; whereas residues F692 (yellow), Q686 (red), and Leu 689 (magenta) are well positioned to directly interact with the IDCL of PCNA.

To better understand the interaction detected between Prox1 and PCNA, we generated several docking models guided by the observation that the interaction is between the PIP box found in the homeo-Prospero domain of Prox1 and the carboxyl-terminus as well as the IDCL of PCNA. Notably, our prior Prox1 structural model [19] predicts that the PIP box of Prox1 folds into a tight 3_10_ helix consistent with the structure of other known PIP box containing PCNA interacting proteins [39-41]. Thus, we tested whether Prox1-PCNA interactions are compatible with the structures of these proteins with the added requirement that the PIP box of Prox1 be contained within the interface region (Figure 4). We also considered whether this interaction could take place without the IDCL, using only the carboxyl-terminus of the PCNA subunit since that was the original hit from the yeast two-hybrid screen. Both predicted models were subjected to a short energy minimization cycle (2,000 steps) using AMBER. The minimized models were then compared by their total and percent buried surface area.

In a trimeric PCNA-Prox1 complex, 2150Å^2^ of surface area is buried whereas only 1720 Å^2^ is buried for the carboxyl-terminal PCNA/Prox1 complex. However, although the absolute buried surface area is higher with trimeric PCNA/Prox1, it only corresponds to roughly 20% of the entire complex surface area; whereas, in the case of the carboxyl-terminal PCNA/Prox1 complex, nearly 28% of the entire surface area is buried. Our models do not explain why amino acids 135–261 of PCNA interacted less efficiently with Prox1 than the original amino acids 167–261 clone in yeast two-hybrid experiments (Figure 2), however, this may reflect destabilization of the chimeric protein structure compared to either the shorter or longer construct. Notably though, the predicted interaction between Prox1 and PCNA will preserve accessibility between Prox1 and DNA. Overall, these models support the idea that Prox1 interacts with PCNA via the PIP box.

### PCNA and Prox1 colocalize in the lens epithelium and developing liver

**Figure 5:**
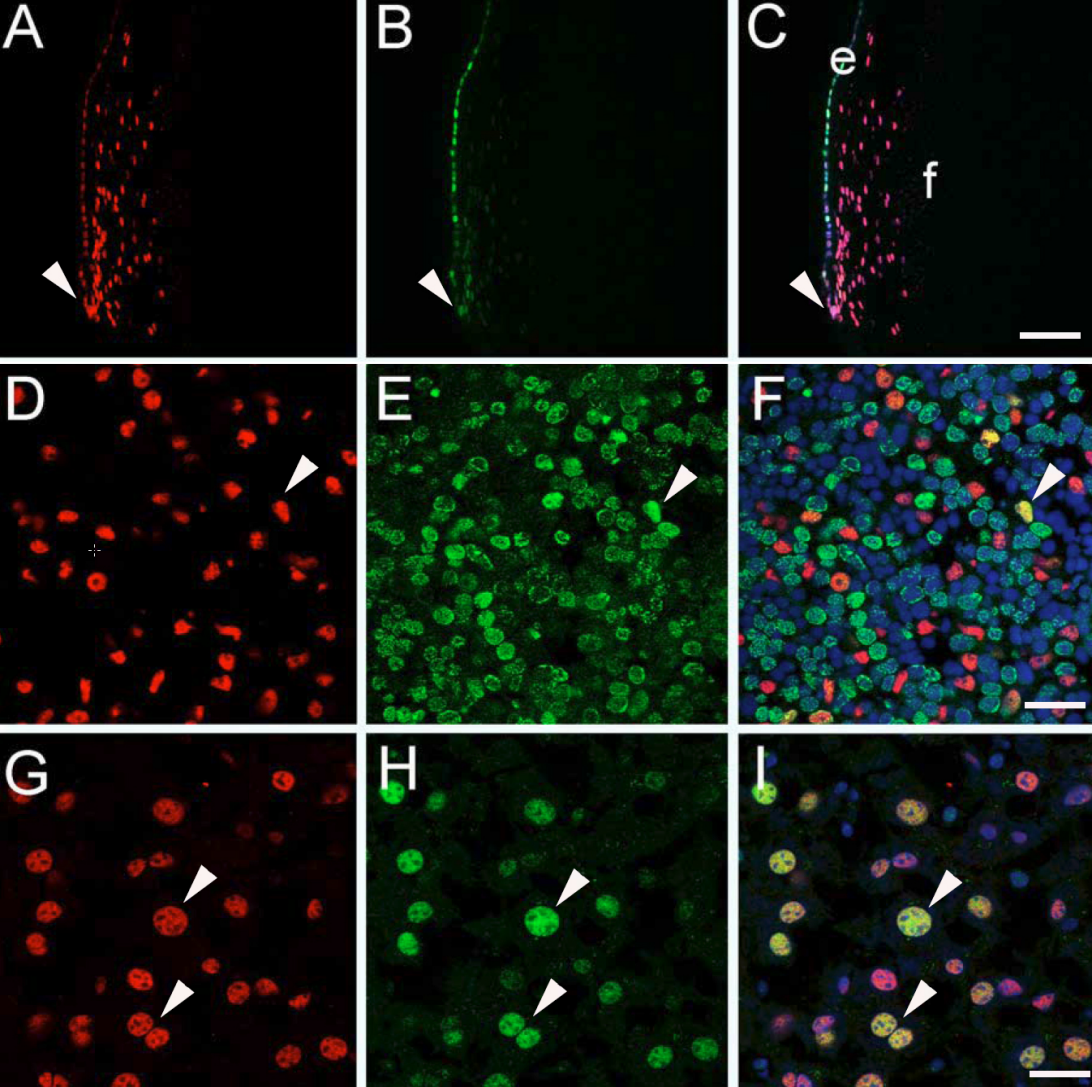
Co-localization of Prox1 and PCNA in Prox1 expressing tissues. **A**-**C**: Adult mouse lens, (**A**) Prox 1 (red) is found in the nuclei of all lens cells, although the relative amounts are higher in the lens fiber cells. **B**: In contrast, PCNA (green) is found at variable levels in the transition zone and sharply downregulates early in fiber cell differentiation. **C**: The overlap between the Prox1 and PCNA signals (white or yellow) shows that these molecules have the potential to interact in the lens epithelium, particularly as the epithelial cells are making the fate decision to become fiber cells (arrowhead). Scale bar=77 μm. **D**-**F**: 16.5 days post coitum (dpc) mouse liver, (**D**) Prox1 (red) is found in a subset of embryonic liver cells. **E**: PCNA (green) is expressed by almost all cells of the embryonic mouse liver. **F**: High levels of PCNA (green) and Prox1 (red) are not typically co-localized, but such co-localization (arrowhead) is found in some cell nuclei (yellow). Scale bar=24 μm. **G**-**I**: Adult mouse liver, (**G**) Prox1 (red) is found in a subset of adult liver cells. **H**: PCNA (green) is only found in a small subset of adult liver cells. **I**: High levels of PCNA (green) and Prox1 (red) are usually co-localized (arrowhead) in cell nuclei (yellow). Scale bar=24 μm. Blue signal in panels **C**, **F**, and **I** is the DNA stain DraqV.

To test whether PCNA-Prox1 interactions could be biologically relevant, immunolocalization for these proteins was performed on two known Prox1 dependent tissues, the lens and the liver (Figure 5D-I). In the adult lens, Prox1 is expressed uniformly in the equatorial epithelium where it co-localizes with PCNA, although PCNA staining intensity was variable cell to cell. As Prox1 levels increase at the onset of lens fiber cell differentiation (arrowhead), PCNA levels decline sharply (Figure 5A-C). In the embryonic mouse liver, Prox1 positive cells are scattered through the tissue. While most embryonic liver cell nuclei are positive for PCNA, occasional Prox1 positive nuclei also express abundant PCNA (Figure 5D-F; arrowhead). In the adult liver, most cells exhibiting strong PCNA signals were also strongly positive for Prox1 (Figure 5G-I; arrowhead).

### PCNA repressed Prox1 mediated transcriptional activation of the chicken βB1-crystallin promoter

**Figure 6:**
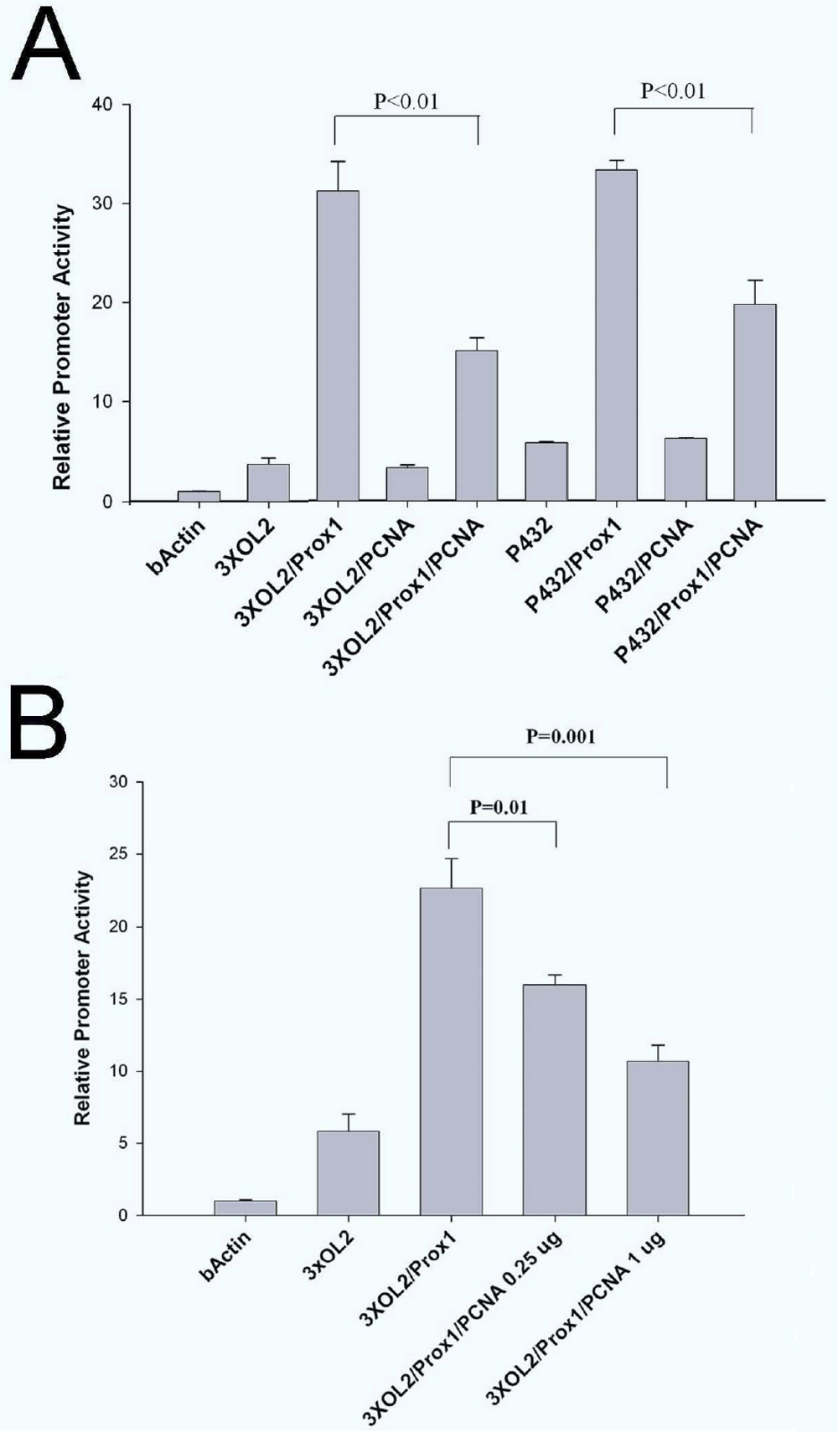
PCNA repressed Prox1 transactivation of the chicken βB1-crystallin promoter in transfection assays. **A**: Prox1 activated the expression of reporter constructs consisting of either the 3XOL2 element cloned upstream of the β-actin minimal promoter or the full length chicken βB1-crystallin promoter (−432/+30). While PCNA did not have a measureable effect on either construct alone, cotransfection of the Prox1 and PCNA expression vectors resulted in a significant repression of Prox1 mediated activation. **B**: Increasing the amount of PCNA expression vector to 1 μg resulted in more robust transcriptional repression. P values were calculated using Student’s *t*-test.

Prox1 is a known transcriptional activator of the full length lens fiber cell specific chicken βB1-crystallin promoter and can also activate an artificial construct composed of multimers of the Prox1 site found at −76 of this promoter (OL2) fused to the β-actin minimal promoter [17,19]. To test whether PCNA can affect Prox1 function as a transcriptional activator, we cotransfected Prox1 and PCNA expression vectors with both full length βB1-crystallin promoter and OL2 reporter constructs. As previously reported, Prox1 activated both the full length βB1-crystallin promoter as well as a vector consisting of three consensus Prox1-responsive OL2 elements placed upstream of the β-actin basal promoter linked to the CAT reporter gene (Figure 6A). Transfection of a PCNA expression vector alone did not significantly affect the activity of either reporter, however, cotransfection of PCNA with Prox1 significantly repressed Prox1 activation of both reporter constructs (Figure 6A). To test how the ratio of Prox1 to PCNA modulates this effect, we also performed cotransfections with a larger amount of PCNA expression plasmid than previously and found that PCNA repressed Prox1 mediated transcription more potently (Figure 6B).

## Discussion

Prox1 is essential for the development of multiple tissues including the lens [1], retina [4], liver [3], pancreas [5], and lymphatic system [2]. Notably, in some cases such as the lens, Prox1 is necessary for cell cycle exit and the terminal differentiation [1]. In contrast, Prox1 is required for the proliferation of the lymphatic vasculature during development [2,42] and overexpression of Prox1 is associated with elevated invasiveness of Karposi’s sarcoma [10]. Further, Prox1 is required for cell proliferation of hepatoblasts during liver development [8], but loss of Prox1 from hepatocytes is associated with the development of hepatocellular carcinoma [7] showing that Prox1 can play different roles at different developmental stages in a single organ system. Similarly, while Prox1 is accepted to function as a transcription factor, its roles are complex since it can function as a transcriptional corepressor of nuclear hormone receptors [15] as well as a direct DNA binding transcriptional activator or repressor [18,19]. In fact, Prox1 repressive and activating sites have both even been detected in the promoter of a single gene [19].

### Prox1 interacts with PCNA through the PIP box

To gain insight into the mechanisms underlying these diverse functions of Prox1, we screened an 11.5 dpc mouse embryo yeast two-hybrid library with the carboxyl-terminus of Prox1 which includes the DNA binding domain and identified proliferating cell nuclear antigen (PCNA) as a Prox1 interacting partner. PCNA is best known as a molecule essential for nuclear processes associated with the cell cycle including DNA replication, DNA repair, cell cycle control, and chromatin remodeling [43]. Native PCNA forms a homo-trimeric ring shaped complex with sixfold symmetry in which the outside surface is composed of β-sheets and parallel α-helices [44]. PCNA in this structure can encircle double strand DNA and serves as a “sliding clamp” which stabilizes the interaction of DNA polymerases and other replication factors with DNA during replication [23]. However, PCNA interacts with a wide variety of other proteins as well, including cyclins, cyclin dependent kinases, and various components of the DNA damage repair machinery [44].

Notably, most known PCNA protein partners interact with the interdomain connecting loop (IDCL, amino acids 119–133) of PCNA via a conserved PCNA interacting signature sequence known as the PIP box although they make additional stabilizing contacts outside of this region [23]. Consistent with this, we identified a consensus PIP box in the Prospero domain of Prox1 (Figure 3) which was required for interactions between the homeo/Prospero domain of Prox1 and PCNA. Computer modeling found a good geometric fit between this region of Prox1 and PCNA, with most of the contacts detected between the PIP box of Prox1 and the IDCL/carboxyl-terminus of PCNA. Notably though, the original PCNA clone identified in our yeast two hybrid screen lacks the IDCL domain and interacts with Prox1 more avidly than the carboxyl-terminus with the IDCL included. Our modeling results suggest that Prox1 interactions with trimeric PCNA in the absence of DNA would occur via the IDCL. A notable feature of the PIP-box is that it folds into a tight 3_10_ helix with hydrophobic residues facing the surface while the IDCL of PCNA presents a rather linear extension of non-polar residues. These features make it possible for PCNA interacting proteins to bind PCNA by inserting this hydrophobic key into the non-polar pocket formed by the IDCL [40,45,46]. However, it has been reported that some proteins interact with the IDCL of PCNA in the absence of DNA while they interact primarily with the C-terminus of PCNA when PCNA encircles double strand DNA due to conformation changes in PCNA upon DNA interaction [47-49].

Recently it has been reported that PCNA may also play roles in transcription as well as DNA replication/repair. PCNA interacts with P300 [26] and PCNA interactions with HDAC1 promote chromatin hypoacetylation and transcriptional repression [50]. PCNA also interacts directly with the DNA binding domain of the retinoic acid receptor and represses its transcriptional activity [25] while PCNA interactions with unliganded estrogen receptor α (ERα) result in stabilized ERα DNA binding and transcriptional activation. Here we showed that PCNA can also repress Prox1 mediated activation of both the full length chicken βB1-crystallin and the Prox1 activatable element, OL2, in transfection assays. However, PCNA did not significantly affect Prox1 mediated repression of the −290 and −220 elements [19] of the chicken βB1-crystalin promoter (data not shown). These data suggested that PCNA can modulate Prox1 function as a transcriptional activator but not its repressive roles. Interestingly, it was previously reported that Prox1 overexpression in blood vessel endothelial cells upregulates the expression of PCNA and other cell cycle related genes leading to phenotypic conversions of these cells to lymphatic endothelium [11]. This suggests that in some systems Prox1 and PCNA could act in a feedback loop in which Prox1 activates PCNA expression then the elevated levels of PCNA repress Prox1 mediated transcriptional activation.

The lens is composed of two cell types, the anterior epithelial cells and the posterior fiber cells [51]. In the adult lens, the central portion of the lens epithelium does not usually proliferate except in response to injury, while the equatorial epithelial cells continue to divide throughout life, albeit slowly. In contrast, lens fiber cells do not divide and in most circumstances are incapable of mitosis [52-54. Prox1 is found in the nuclei of all lens cells, although its levels are relatively higher in the lens fibers which require Prox1 for their differentiation [37] (Figure 5). In contrast, PCNA is detected throughout the lens epithelium, even in cells unlikely to be actively cycling while its levels decrease sharply as lens fiber cells begin to differentiate (Figure 5). It should be emphasized that in the adult lens, PCNA is detected in epithelial cells unlikely to be progressing robustly through the cell cycle suggesting that it is performing other functions besides acting as a sliding clamp for DNA polymerase. The presence of PCNA in these cells may be due to both its long half life [43] and a function in transcriptional control.

Overall, our data suggest that PCNA binding to Prox1 can diminish Prox1’s ability to function as a transcriptional activator. We have previously proposed that Prox1 interactions with the high affinity Prox1 binding sites in the chicken βB1-crystallin promoter can lead to Prox1 mediated repression of this gene when Prox1 is present at low levels [19]. In contrast, Prox1 interactions with the lower affinity sites present in the same promoter results in transcriptional activation of the crystallin gene as Prox1 levels increase during lens fiber cell differentiation. The present data suggest that PCNA-Prox1 interactions in the lens epithelium could further block the Prox1 present in the lens epithelium from inappropriately driving lens fiber cell differentiation. Then, as PCNA levels decrease during early fiber cell differentiation, this transcriptional attenuation would be relieved and Prox1 would be capable of driving the formation of lens fiber cells.
